# Robot-assisted adrenalectomy: state of the art

**DOI:** 10.1007/s13304-020-00915-2

**Published:** 2020-11-11

**Authors:** Gabriele Materazzi, Leonardo Rossi

**Affiliations:** Department of Surgical, Medical and Molecular Pathology and Critical Area, Endocrine Surgery Unit, University of Pisa, Pisa Hospital, Via Paradisa 2, Edificio 30 J, 56100 Pisa, Italy

**Keywords:** Robotic, Adrenalectomy, Adrenal gland, Laparoscopic, Retroperitoneal

## Abstract

Currently, laparoscopic adrenalectomy is worldwide considered the gold standard technique. Both transperitoneal and retroperitoneal approaches have proved their efficacy with excellent outcomes. Since the introduction of da Vinci System (Intuitive Surgical, Sunnyvale, CA), robotic surgery has made many steps forward gaining progressively more diffusion in the field of general and endocrine surgery. The robotic technique offers advantages to overcome some laparoscopic shortcomings (rigid instruments, loss of 3D vision, unstable camera). Indeed, the robotic system is provided of stereoscopic 3D-magnified vision, additional degree of freedom, tremor-filtering technology and a stable camera. Recently, several case series have demonstrated the feasibility and the safety of robot-assisted adrenalectomy in high-volume centers with outcomes comparable to laparoscopic adrenalectomy. Notwithstanding, the technical advantages of the robotic system have not yet demonstrated significant improvements in terms of outcomes to undermine laparoscopic adrenalectomy. Moreover, robotic adrenalectomy harbor inherits drawbacks, such as longer operative time and elevated costs, that limit its use. In particular, the high cost associated with the use of the robotic system is primarily related to the purchase and the maintenance of the unit, the high instruments cost and the longer operative time. Notably, these aspects make robotic adrenalectomy up to 2.3 times more costly than laparoscopic adrenalectomy. This literature review summarizes the current available studies and provides an overview about the robotic scenario including applicability, technical details and surgical outcomes.

## Introduction

The first successful laparoscopic adrenalectomy (LA) was described by Gagner et al. in 1992 [1]. Although initially adopted for the management of small benign lesions, currently LA is worldwide considered the gold standard technique for the treatment of functional and non-functional adrenal lesions with reported masses up to 11–12 cm in size [2–4]. LA has proved to be safe and should be preferred to open adrenalectomy due to shorter length hospital stay, lower blood loss and equivalent morbidity, except for selected cases, such as malignant tumors potentially infiltrating surrounding organs [4]. Nonetheless, recently the frequency of minimally invasive surgery for adrenocortical carcinoma is increasing although further studies are needed to define its role in the treatment of malignancy [5, 6]. Further, the superiority of LA over the open approach was sustained even for benefits derived from a reduced post-operative pain and wound complications and better cosmesis [7].

Additionally, a retroperitoneoscopic technique with the patient placed in prone position was proposed as alternative approach and gained popularity, thanks to the excellent outcomes demonstrated by Walz et al. [8]. An important benefit of the posterior approach is the direct access to the operative field avoiding the need for mobilization of adjacent structures [9]. Further, the retroperiteoscopic technique reduces the risks of complications associated with entering in the peritoneal cavity and displayed some technical benefits for patients with bilateral adrenal lesions or in patients prior underwent abdominal surgery with potential adherences [10]. On the other hand, some limitations were represented by the narrow working space, which could collapse in case of excessive suction, close position of the trocar and reduced angulation of the rigid laparoscopic instrument [9].

After the approval of the Robotic system for the surgical scenario, it has gained progressively more diffusion. First robotic-assisted adrenalectomy (RA) was performed by Piazza et al. in 1999 [11] for a patient with right adrenocortical adenoma using the ZEUS AESOP 2000.

Since then, many steps forward have been made and with the introduction of da Vinci System (Intuitive Surgical, Sunnyvale, CA); the robot-assisted adrenalectomy has received so much attention that has been added to surgical options, although LA still remains the gold standard technique.

However, the robotic technique offers advantages to overcome some laparoscopic shortcomings (rigid instruments, loss of 3D vision, unstable camera). Indeed, the robotic system is provided of stereoscopic 3D-magnified vision, additional degree of freedom by means of multi-articulated robotic arms with Endowrist technology, and a stable camera. Further, the natural hand tremor is eliminated and even the position is made more ergonomic, thanks to a comfortable sitting [12].

Despite these technical advantages, RA has not yet demonstrated significant improvements in terms of outcomes to undermine LA and consequently concern about robotic system has raised. First, even for experienced laparoscopic surgeons, a significant learning curve is needed (about 20 cases) to obtain results comparable to the standard [13]. Moreover, due to the additional steps required, such as the docking of the robotic tower, a prolonged operative time is acknowledged [12, 14]. Last but not least, the major drawback of the robotic surgery is represented by the elevated costs of the purchase and for the maintenance of the unit [15, 16].

The present literature review summarizes the current available studies and provides an overview about the robotic scenario including applicability, technical details and surgical outcomes.

## Surgical technique

### Transperitoneal approach

The patient is placed in lateral decubitus position on the opposite side of adrenal pathology. After the pneumoperitoneum is established, three to four robotic ports (on the right side an additional trocar for liver retraction is required) are generally placed two fingerbreadths below the costal margin. An optional first assistant port placed near the rectus muscle border is sometimes necessary for retraction or suction [17].

Docking of the robotic system and connection of the robotic arms.

On the right side, after insertion of the robotic instruments, a liver retractor is introduced through the medial port by the assistant to provide a gentle medial traction on the underside of the liver to provide exposure of the retroperitoneum.

The right hepatic triangular ligament is dissected and subsequently the right hepatic lobe is freed from the diaphragm. Care is taken not to proceed too far medially and injury the right hepatic vein. The retroperitoneum is incised near the border of the inferior vena cava and the adrenal vein is identified, closed and divided. Accurate dissection in this area is of paramount importance as bleeding from the cava can be significant. The peritoneal layer overlying the adrenal gland is then grasped and used as a handle to provide tension in the upward direction. Approaching from the inferior aspect, the dissection proceeds in the relatively avascular plane beneath the adrenal gland until its complete mobilization. Once the specimen is freed from its remaining attachments, it is removed by means of an endocatch bag through an enlarged trocar port [17].

On the left side, first step consists in dissecting of the spleno-colic ligament. Subsequently, the colon in mobilized caudally and the spleen medially. Caution should be used when retracting the spleen due to the lack of haptic feedback with the robot. Mobilization of the spleen continues medially until the spleen will lie under its own weight. Often, when progressing through this avascular plane, the tail of the pancreas and splenic artery are encountered: these structures may need to be gently dissected free and mobilized in continuity with the spleen to allow adequate exposure to the adrenal gland. To obtain an optimal access to the adrenal area, dissection is continued up until the diaphragm and the great curvature of the stomach comes into view. Dissection along the medial plane in the groove between the adrenal gland and the pancreas can help to isolate the area of the adrenal vein. Hence, the adrenal vein is closed and divided and the operation proceeds following the same steps of contralateral procedure [17].

### Retroperitoneal approach

The patient is placed on a prone jackknife position on a Wilson frame. The initial entry to the Gerota’s space is obtained through a 12–15 mm incision at the end of the tip of the 12th rib using an optical trocar. The space is initially created using a dissecting balloon or with digital exploration. Two to three additional 8- or 12-mm trocars are also inserted, under the direction of a finger place in the retroperitoneum, 1–2 cm apart from the tip of the ribs to prevent subcostal nerve injuries and in a position which avoids collision [9, 18].

The operating space is developed by insufflating with carbon dioxide to a pressure between 15 and 24 mm Hg. In general, lower pressures are initially used and increased only high enough to provide an adequate operative working field.

Docking of the robotic system and connection of the robotic arms. The dissection begun using the upper pole of the kidney as the initial anatomic landmark. The adrenal vein is identified and early divided.

A careful blunt retraction of the adrenal gland can be performed to dissect the surrounding fat and eventual attachments. Once dissection is completed, the specimen is removed by means of an endocatch bag through a trocar port [9, 18].

## Outcomes

### Complications and conversion rate

To evaluate the safety of a novel technique, it is essential to analyze the complications and conversion rate and to compare them with those of alternative techniques.

Although data reported in literature are not homogeneous and the results vary widely due to lacking in many studies of a classification for severity of complication, and since not always the distinction between intra-operative and post-operative complication is clarified, overall, RA is reported to be safe with a low complications rate [4, 14]. Further, the majority of postoperative morbidity included Clavien 1 and 2 complications, whereas severe complications leading to reoperation are unusual [7].

Taking into consideration studies with more than 5 patients, morbidity varied from 0 to 27.2% (*see *Table 1). In a recent large case series of more than 300 patients [19], it has been reported a post-operative complication rate of 9.2%; reoperations for severe complications (Clavien > 3b) were required in only 1% of cases. These data are substantially superimposable to whom reported by large series of LA [20–22].

**Table 1:** Robot-assisted total adrenalectomy series

Authors	Cases	Approach (TP/RP)	EBL (ml)	MOT (min)	MTS (cm)	Intra-operative complications	Post-operative complications	Conversions	Re-operations	Mean hospital stay (days)	Mortality
Giulianotti et al. [27]	42	42/0	27	118	5.5	1 capsular tear (2.4%)	1 clostridium infection (2.4%)	0	0	4	1 (2.4%)
Morelli et al. [39]	41	41/0	NR	176.6	4.9	2 vascular lesion (4.9%)	1 pneumonia (2.4%) 1 supraventricular tachyarrhithmya (2.4%)	0	0	3.3	0
Quadri et al. [54]	43	43/0	84.8	127.1	5	0	2 clostridium difficile infection (4.7%) 1 pneumonia (2.3%) 1 adrenal failure (2.3%)	1 (2.3%)	0	3.6	0
Morino et al. [28]	10	10/0	NR	169	3.3	2 intra-operative hypertension (20%)	0	4 (40%)	0	5.4	0
Greilsamer et al. [19]	303	303/0	NR	88.9	3.6	9 capsular tear (3%)	8 incisional hernia (2.6%) 5 abdominal abscess (1.7%) 1 renal infarction (0.3%) 2 hematoma (0.7%) 1 paresthesia (0.3%) 4 chronic pain (1.3%) 3 pneumonia (1%) 2 urinary infection (0.7%) 1 facial oedema (0.3%) 1 hypotension (0.3%)	9 (3%)	3 (1%)	5.5	0
Brandao et al. [34]	30	30/0	50	120	3	1 NR (3.3%)	2 bleeding (6.7%) 1 nausea/vomiting (3.3%) 1 hyponatiremia (3.3%) 1 atrial fibrillation (3.3%) 1 wound infection (3.3%)	0	1 (3.3%)	2	0
Raffaelli et al. [42]	26	13/0	NR	221.5	NR	3 bleeding (23.1%)	1 respiratory failure (14.4%) 1 collection (14.4%)	0	0	4.4	0
You et al. [36]	15	15/0	NR	207	2.6	0	1 chylous ascites (6.7%) 1 wound seroma (6.7%)	0	0	5.9	0
Agcaoglu et al. [52]	66	66/0	60,4	113.9	4.3	3 bleeding (4.5%)	0	3 (4.5%)	0	2.6	0
Wu et al. [26]	5	5/0	90	188	5.1	0	0	0	0	4	NR
Raman et al. [82]	40	40/0	48,9	116.7	7	1 splenic hematoma (2.5%) 1 bile tract injury (2.5%)	1 hyponatremia (2.5%) 1 hemiparesis (2.5%)	4 (10%)	0	3.2	0
Pahwa et al. [83]	25	25/0	85	139	4.5	2 hypertension (8%)	1 hypotension (4%) 2 pleural effusion (8%)	0	0	2.5	0
Ludwig et al. [18]	6	0/6	55	135	2.8	0	0	1 (16.7%)	0	1.3	0
Winter et al. [78]	30	30/0	NR	185	2.4	0	1 ileus (3.3%) 1 hypoxemia (3.3%)	0	0	2	0
Brunaud et al. [30]	100	100/0	NR	99	2.9	1 capsular tear (1%) 3 bleeding (3%)	3 pneumonia (3%) 2 urinary tract infection (2%) 2 wound infection (2%) 1 anemia (1%) 1 hematoma (1%) 1 facial oedema (1%)	5 (5%)		6.4	
Undre et al. [84]	2	2/0	150	149.5	1.3	0	1 pulmonary embolus (50%)	0	0	4	0
Bentas et al. [85]	4	4/0	< 100	220	3.8	0	0	0	0	5	0
Nordestrom et al. [25]	100	100/0	NR	113	5.3	2 bleeding (2%) 1 atrial fibrillation (1%)	2 fever (2%) 2 bleeding (2%) 2 atrial fibrillation (2%) 1 urinary catheter (1%) 2 adrenal failure (2%) 1 confusion (1%) 1 hypotension (1%) 1 paroxysmal tachycardia (1%) 1 subcutaneous emphysema (1%)	2 (2%)	0	NR	0
D'Annibale et al. [38]	30	30/0	< 50	231.1	5.1	1 capsular tear (3.3%) 1 marked arterial instability (3.3%)	1 abdominal hematoma (3.3%) 1 pneumonia (3.3%) 1 myocardial infarction (3.3%)	1 (3.3%)	0	5.2	0
Krane et al. [86]	5	5/0	< 25	75.7 (OCT)	5.3	0	0	0	0	1.25	0
Akarsu et al. [87]	10	10/0	< 50	98	5.4	1 diaphragm injury (10%)	0	0	0	4.1	0
Desai et al. [88]	2	2/0	75	137.5	3.8	1 capsular tear (50%)	0	0	0	2.5	0
Kahramangil et al. [89]	200	116/84	13.9	147	3.8	0	3 urinary tract infection (1.5%) 1 pancreatic leak (0.5%) 1 pneumonia (0.5%) 1 ileus (0.5%) 1 heart failure (0.5%) 1 axillary neuropathy (0.5%) 1 surgical site abscess (0.5%)	5 (2.5%)	0	1	0
Probst et al. [79]	28	28/0	NR	128.5	5.4	1 blood pressure dysregulation (3.6%) 1 spleen injury (3.6%)	2 NR (7.2%)	0	2 (7.2%)	6.8	0
Feng et al. [90]	3	0/3	NR	136.3	4.6	0	0	0	0	NR	0
Agcaoglu et al. [35]	31	0/31	25.3	163.2	3.1	0	0	0	0	1	0
Lairmore et al. [10]	17	0/17	46.5	177.3	2.4	0	1 chest pain (5.9%) 1 hematoma (5.9%)	1 (5.9%)	1 (5.9%)	1.5	0
Kim et al. [91]	74	0/74	NR	111.6	2.6	0	0	0	0	4.1	0
Berber et al. [9]	8	0/8	24	214.8	2.9	0	0	0	0	1	0
Makay et al. [92]	76	NR	83	127	4.3	0	4 incisional hernia (5.3%) 2 urinary tract infection (2.6%) 2 chronic pain (2.6%) 1 arrhythmia (1.3%) 1 atelectasis (1.3%)	3 (3.9%)	NR	2.4	0
Colvin et al. [93]	20	7/13	59.4	130.2	1.7	0	0	0	0	1.1	0
Agcaoglu et al. [54]	25	18/7	83.6	159.4	6.5	0	0	1 (4%)	0	1.4	0
Aliyev et al. [37]	26	18/8	36	149	5.5	0	0	0	0	1.2	0
Pavan et al. [67]	81	75/5	50	150	2.7	5 NR (6.2%)	17 NR (21%)	2 (2.5%)	NR	2	NR
Fang et al. [32]	41	41/0	173	210	6.2	NR	11 NR (26.8%)	0	NR	3	0
Aksoy et al. [40]	42	28/14	50.3	186.1	4	0	1 urinary tract infection (2.4%) 1 pneumothorax (2.4%)	0	0	1.3	0
Pineda Solis et al. [31]	30	NR	30	189.7	3.2	0	0	0	0	1.9	0
Feng et al. [94]	85	69/16	21.3	133.2	3.7	NR	NR	NR	NR	1.7	NR
Karabulut et al. [33]	50	32/18	41	166	3.9	0	1 atelectasis (0.5%)	1 (0.5%)	0	1.1	0

*NR* Not Reported, *TP* Trans-peritoneal, *RP* Retro-peritoneal, *EBL* Estimate blood loss, *MOT* Mean operative time, *MTS* Mean tumor size, *OCT* Only console time

Moreover, Greilsamer et al. [19]reported four independent factors for intra-operative and post-operative complications: history of previous ipsilateral surgical procedure, tumor size, patient’s age, and need of conversion. In particular, regarding previous abdominal surgery, this datum is in contrast with those reported by a study conducted by Morris et al. [20], which investigates the influence of surgical history on complication rate of LA, without finding any significant association.

The safety of RA is also supported by several meta-analysis which compare the complications and the mortality rate of RA and LA without reporting any significant difference between these two minimally invasive techniques [4, 12, 14, 23, 24].

Worth to be underlined, on the basis of our literature review, robot-assisted adrenalectomy did not harbor any technique specific complication [24–26].

Overall, analyzing data reported in literature, we can extrapolate that RA and LA have similar results in terms of complications and mortality and the incidences of these appear to be mostly related to the pathology or to pre-existent clinical conditions of the patient, rather than to the technique itself, which achieved comparable outcomes [10, 14, 27].

With regard of conversion rate, our review reported values ranging from 0 to 40% (see Table 1). The highest conversion rate was of 40% derived from the first randomized controlled study by Morino et al. [28]. The reasons of conversion were malposition of robotic trocars, prolonged operative time and difficulties to obtain accurate hemostasis. In particular, this last observation realistically was related to the initial lack of advanced energy source with typical use of monopolar scissor or bipolar forceps [29]. Accordingly, excluding this study, the higher conversion rate dropped to 16.5% (see Table 1). Other reported reasons of conversion were local adherence of the tumor to the surrounding organs, technical difficulties and intraoperative bleeding [7]. As a consequence, in some cases, dissection and hemostasis became challenging to manage, leading the surgeon to convert to conventional laparoscopy or open surgery [14]. Furthermore, as reported, the learning curve plays a role in determining the conversion rate [25, 28].

In a recent review on national inpatient sample [16], the authors reported a conversion rate of RA < 2%, a comparable value to that of LA. In agreement with this, several meta-analysis reported similar data between RA and LA regarding the complication rate [12, 14, 23, 24, 29].

Undoubtedly, the Robotic System harbors potential advantages to the surgeon, such as stereoscopic magnified-3D vision, increased instrument dexterity, additional degrees of freedom and more ergonomics, but to date, such features have not evidenced to get superiority compared to LA which remains the current gold standard technique.

These data prompt that RA is feasible, effective and safe but have not shown any advantages in terms of outcomes. Moreover, some concerns still remain regarding the loss of tactile feedback, which is reported to be attributable to the incidence of some complications at the beginning of the experience [30].

### Operative time

Regarding operative times (OT), there is a wide range reported by several studies which varies from 89 to 215 min (see Table 1) [9, 19].

Many comparative studies reported a significant longer operative times for RA vs LA [10, 26, 28, 31, 32] and these data are further confirmed even by recent meta-analysis [12, 14]. Notwithstanding casuistries, with no statistical significant difference in OT between RA and LA, are described too [33–36], the heterogeneity of the results risks to weaken the strength of the reported data. Indeed, the definition of “operative time” is not clarified in large part of the studies, which not defining the starting and the ending of the procedure and, furthermore, only few case series reported separately the docking/set-up time [26, 30, 35, 37]. Moreover, inclusion of transperitoneal along with retroperitoneal approach in the analysis may have further confounded the overall exits. Intuitively, RA harbors a potentially longer operative time attributed to the docking of the robot, the camera calibration, the connection of the robotic arms to the robotic trocar as well as the undocking of the robotic system [12, 23]. These additional steps increase the mean overall “real” operative time by 15–40 min and can be as long as 1 h at the beginning of the experience [7]. Moreover, Agcaoglu et al. [35] reported that the transport of the robotic unit to the operating room, system start-up, draping of the robotic arms and calibrating the robotic camera can take up to 20–25 min.

Additionally, it is reported by several studies that the surgeon’s experience has an impact on influencing the OT [19, 30, 35, 38]. Indeed, Greilsamer et al. [19] reported a significant decrease of the operating time between the first 100 RA and the last 100 RA. Similarly, Agcaoglu et al. [35] reported a significant reduction in the operative time after the 10th RA with retroperitoneal approach, reaching lower values than the laparoscopic counterpart and shortening the skin-to-skin operative time by 28 min. Similar results were reported by Brunaud et al. [30] and D’Annibale et al. [38], who assumed that the additional time may decrease as more procedure are performed and that the plateau of the learning curve is reached, respectively, after 12 and 20 cases. Therefore, Brunaud et al. [30] reported that surgeon’s and first assistant’s experiences, as well as tumor size, play a role in determining the OT of RA.

Overall, it seems reasonable that, beyond the learning curve, since the robotic procedure follows the same surgical steps of the laparoscopic one, these two techniques show a similar “surgical time”; notwithstanding, we have to acknowledge that with the use of robotic system, it is mandatory to take into account even the time needed to dock and undock the robotic tower with its arms. Such steps prolong the overall surgical time from 15 to 60 min, depending on the experience of the team [31].

Moreover, our opinion is that hospitals with dedicated robotic-operating rooms and specific equip are advisable to cut down the additional set-up time.

### Hospital stay and blood loss

Of course, one of the most important advantages of minimally invasive approach is the fast recovery and the short hospital stay (HS). These considerations are true even for RA: basis on our literature review, HS ranges from 1 to 6.8 days (see Table 1).

The length of HS has been evaluated and compared to those of LA in several studies without finding any statistical significant difference [31, 32, 34, 39, 40]. Anyhow, several meta-analysis reported a shorter hospital stay for RA vs LA [4, 10, 12, 14, 23].

Nevertheless, some Authors advocated [41, 42] that the difference in the length of hospitalization could be confounded by many factors and may be not reliable in the comparison between minimally invasive techniques.

Similarly, RA is a safe procedure with a low estimate blood loss (EBL) value (see Table 1). Several case series reported similar EBL values between RA and LA [31, 33, 37]. Consistent with these data, also a recent meta-analysis [12] and a review of a national inpatient sample [16] did not report any significant differences between RA and LA in EBL. Nevertheless, some meta-analysis [4, 14, 29] reported a significant lower EBL in the RA group theoretically attributable to the stereoscopic magnified vision and the more precise dissection. However, worth to focus that the difference, although statistically significant, is not clinically relevant since the gap, as reported in another meta-analysis [43], is very limited (25 ml) and does not influence the patient management or the need of transfusion. According to this, no difference was noted comparing the incidence of blood transfusion between RA and LA [16, 23].

Last but not least, a recent paper by Rothermel et al. [44] reported that blood loss estimations are not a reliable metric to judge surgeons’ performance or patient outcomes.

Overall, RA is undoubtedly safe with optimal outcomes in terms of HS and EBL. Anyway, the potential advantage upon LA—the current gold standard technique—is controversial. In particular, a reduction in the length of the HS can be explained only by a minor trauma [45]. Since the surgical steps of RA and LA are superimposable, and considering that often RA requires additional trocar and is performed in similar surgical times, it seems unreasonable expecting difference in terms of HS. As reported by Agrusa et al. [29], the difference could hide operator relating bias explained with the positive expectation from a novel technique and the medical staff focused on the early hospital discharge.

## Robot-assisted bilateral adrenalectomy

Bilateral adrenalectomy (BA) is required in case of recurrent or persistent Cushing disease, in case of primary bilateral adrenal disease or in case of familial syndromes, such as NEM 2, Von Hippel Lindau Syndrome or congenital adrenal hyperplasia [7]. Robotic surgery is particularly endearing in case of bilateral procedure since it offers a finer dissection and a comfortable seat which can be particularly helpful in case of long procedure [42].

Raffaelli et al. [42], in a retrospective multicenter study involving two university third-care referral centers, compared three different technique for BA (laparoscopic transabdominal adrenalectomy, retroperitoneoscopic adrenalectomy, robot-assisted transabdominal adrenalectomy). Robot-assisted BA was found to have a statistically significant lower number of drains used and shorter hospital stay. Anyway, as clearly explained by the authors, these findings were likely related to the different management protocols used in different centers. Indeed, in one of the involved centers drains are routinely positioned, whereas in the other drains are positioned on the basis of surgeon’s discretion. Further, in one center, patients referred from distance are kept hospitalized until they are able to maintain oral dose of hydrocortisone and can safely travel considerable distance back home, whereas in the other center, patients unable to be maintained under oral dose of hydrocortisone are referred to the endocrinology unit [42].

Since during transperitoneal, BA patients are positioned in lateral decubitus, one of the main drawbacks for this approach is the time duration needed to re-position the patient between the two sides [7, 42]. As in conventional laparoscopy, this limitation is overcome by means of robot-assisted BA with retroperitoneoscopic approach which enables an effortless transition from one gland to the other avoiding repositioning [23, 46]. Moreover, Taskin et al. [46] reported that in case of bilateral macronodular adrenal hyperplasia, since adrenalectomy in these cases can be challenging due to multiple nests of adrenal tissue, the articulated robotic instrument can make the dissection easier compared to the rigid laparoscopic instrument.

Anyway, to date, consistent data of a potential superiority of the robotic over the laparoscopic technique are lacking. Bilateral laparoscopic adrenalectomy has proven to be feasible and safe by several studies [47, 48] and to date, still being the gold standard approach. Overall, the same considerations made for unilateral RA are likely to be valid even for robotic BA.

We believe that the classic retroperitoneoscopic approach has technical advantages in case of BA since it enables to avoid the reposition of the patient leading to shorter operative time with comparable outcomes to transperitoneal approach [48].

Due to the scarcity of comparative papers reported in literature, further studies are required to draw definitive conclusions.

## Robot-assisted adrenalectomy in obese patients

Although the incidence of obesity continues increasing worldwide, the impact of obesity on peri- and post-operative outcomes in patient underwent RA is debatable and available large casuistries are lacking.

Recently, Maker et al. [49] published a video showing a RA in an obese patient (BMI 36 kg/m^2^) concluding that this technique is feasible and safe in obese patient with large tumor.

Moreover, some case series [39, 50] reported that obese patients are good candidates for RA. Brunaud et al. [50] reported no difference in terms of operative time between patients with a BMI > or < to 30 kg/m^2^ underwent RA, whereas a significant higher operative time was registered for patients > 30 kg/m^2^ underwent LA.

Furthermore, Greilsamer et al. [19], in a study involving a large sample of obese patients (82), determined that obesity was not significantly associated with longer operative time or peri-operative complications. Notwithstanding, a recent study by Pedziwiatr et al. [51] conducted on 520 patients underwent LA and subdivided on the basis of BMI in four groups (normal weight; overweight; obese; morbidly obese) reported similar results. Indeed, the authors did not found any statistical significant difference between groups in terms of operative time, complications, conversions and blood loss. The authors concluded that LA is feasible regardless of BMI and that obesity has no influence on short-term outcomes after transperitoneal LA [51].

Additionally, Agcaoglu et al. [52] in a study focused on the evaluation of the impact of BMI on outcomes of RA, observed no statistically significant differences between two groups of patients divided on the basis of BMI (< or > 30 kg/m^2^) in terms of operative time, complications, conversions, blood loss and length of hospitalization.

A comparative prospective study on obese patients was performed in 2013 by Aksoy et al. [40]. The authors included in this paper 42 obese patients who underwent RA and 57 obese patients who underwent LA. They found no statistically significant differences between the two groups, concluding that in adrenal surgery, the benefits provided by articulated instruments and 3-D magnified vision proper of the robotic surgery are nullified in obese patients for the difficulties in maintaining exposure and dissection.

In conclusion, peri-operative outcomes in obese patients who underwent RA are still debated and controversial, but we believe that in experienced hands the robotic technique can be performed safely with results comparable to those obtained in non-obese patients. Nonetheless, same outcomes are achievable with LA, which currently remains the gold standard technique.

## Robot-assisted adrenalectomy in patients with large tumor

Minimally invasive resection of large adrenal masses has raised two major concerns: first, the risk of capsular disruption or incomplete removal of an unsuspected malignant tumor with increased risk of recurrence; second, the technical difficulty in dissection due to the large size of the lesion [41, 53].

Several studies investigate the feasibility and the safety of minimally invasive adrenalectomy for large tumors [39, 54, 55]. In 2012, Agcaoglu et al. [54] conducted a comparative study between RA and LA performed for large adrenal masses. They found that RA leads to a significantly shorter operative time and hospital stay with a lower rate of conversion to open surgery, and claimed that the robotic approach has become their preferred minimally invasive technique for removing large adrenal masses.

Quadri et al. [55] in a recent study conducted on 43 cases of RA, divided patients on the basis of tumor size (< and > of 5 cm) and reported no significant differences in terms of operative time, length of hospital stay, readmission, complications and conversion rate. Extending the size limit to 8 cm, the only statistically significant difference was in terms of higher conversion rate. Anyway, due to the small sample (only six patients), larger studies are needed to better assess differences in peri-operative and post-operative outcomes in tumors > 8 cm versus < 8 cm.

In a systematic review, Teo et al. [41] concluded that RA is feasible in patient with large tumors and they suggested that the good outcomes obtained with the robotic approach can be due to the articulated wristed instruments and the 3-D magnified vision which enable a faster and more accurate dissection. Moreover, they recommended the transperitoneal approach since the restricted retroperitoneal space can make the dissection challenging with large lesions.

Thompson et al. [56] in an analysis of Swedish database including 659 adrenalectomies (37.9% were RA) reported that the robotic approach was preferably used in patients with large tumors, suggesting some advantages of the robotic system in these cohort of patients.

Anyway, laparoscopic adrenalectomy for large tumor has previously described to have good outcomes [57]. Conzo et al. [58] in a recent systematic literature review reported that in selected cases LA is feasible and safe even for large masses (> 6 cm) and that the retroperitonescopic approach is demonstrated feasible for tumors up to 8 cm.

In conclusion, our hypothesis is that skilled surgeons can performed safely both LA and RA, in patients with large tumors, with good outcomes. Notwithstanding, it is mandatory to adhere to oncologic principles and we believe that large adrenal lesions should be managed in high-volume centers and that suspected adrenal carcinomas are preferably approached by open technique. Overall, the robotic system shows endearing features for large lesions, but further studies are required for the assessment of its impact on outcomes and to evaluate if these qualities outweigh the burden of the costs.

## Robot-assisted partial adrenalectomy

Although the standard of care for the major part of adrenal pathologies is total adrenalectomy, there are a few situations in which partial adrenalectomy is an option. Indeed, adrenal-sparing technique plays a role in patients with bilateral adrenal lesions or a mass within a solitary adrenal gland or in patients at the highest risk of developing multiple adrenal tumors, such as in hereditary diseases.

Partial adrenalectomy (PA) has the advantage of preserving adrenocortical function and, if successful, avoiding steroid supplementation [59]. In a literature review, Kaye et al. [60] reported that the number of parenchyma-sparing adrenalectomy is increasing and surgical outcomes and peri-operative complications are similar to those reported for total adrenalectomy.

Robot-assisted partial adrenalectomy was first described in 2006 by St Julien et al. [61] in a patient with bilateral pheochromocytoma and Von Hippel Lindau disease. The robotic system seems to be well-suitable for this technique since it enables a particularly delicate manipulation, thanks to the articulated wristed arms with multiple degree of freedom and to the 3D-magnified vision.

Several studies reported the feasibility and the effectiveness of robotic-partial adrenalectomy (see Table 2). Recently, Ye et al. [59] reported their experience for partial adrenalectomy with transperitoneal approach on 13 patients with optimal outcomes. Indeed, beyond the good technical results (low operative time and blood loss), they reported no conversions and no post-operative complications. At a median follow-up of 12 months, no patients required steroid replacement and no recurrences occurred [59].

**Table 2:** Robot-assisted partial adrenalectomy series

Authors	Cases	Approach (TP/RP)	EBL (ml)	MOT (min)	MTS (cm)	Steroid replacement	Local recurrence	Conversions	Mean hospital stay (days)	Mortality
Ye et al. [59]	13	13/0	20	75	3.3	0	0	0	2.3	0
Asher et al. [62]	15	15/0	161	163	2.7	1 (6.7%)	0	1 (6.7%)	NR	0
Simone et al. [64]	10	10/0	150	65	1.8	NR	0	0	3	0
Boris et al. [63]	13	13/0	150	200	2.7	1 (7.7%)	0	1 (7.7%)	NR	0
Gupta et al. [65]	4	4/0	97.5	77.5	4.5	NR	0	0	4	0

*NR* Not Reported, *TP* Trans-peritoneal, *RP* Retro-peritoneal, *EBL* Estimate blood loss, *MOT* Mean operative time, *MTS* Mean tumor size

Moreover, Asher and colleagues [62] reported the largest series of 15 cases of PA (on 12 patients). All of the patients were affected by pheochromocytoma. They reported only one conversion and one complication; further, no intra-operative hypertensive or tachyarrhythmics events were registered and after a median follow-up of 17.3 months, there were no recurrences and only one patient with a solitary gland needed steroid replacement. Similarly, other casuistries showed similar results [63–65]. Interestingly, Kohene et al. [66] reported a case of a patient who underwent robot-assisted partial adrenalectomy with retroperitoneoscopic approach.

Simone et al. [64] claimed that the main advantages of robot-assisted partial adrenalectomy are the minimal manipulation of the surrounding adrenal cortex and the preservation of blood supply of the adrenal remnant tissue, steps which are particularly challenging with the laparoscopic approach.

Despite encouraging results, however, only few case series are reported in literature with small samples and there does not exist any comparative study between robotic- and laparoscopic-PA. Anyway, the robot-assisted PA may offer some technical benefits although further investigations are needed to proper define the role of this approach in adrenal-sparing procedure.

## Robot-assisted single-site adrenalectomy

Recently, laparoendoscopic single-site (LESS) adrenalectomy has been introduced on the basis that with a smaller number of incisions, enhancement of cosmesis and reduction of associated port site complications can be obtained [41]. Further, patients predisposed to a delayed wound healing, such as Cushing’s patients, may avoid wound complications [41]. The drawbacks of LESS consist in a smaller distance between instruments and in the loss of their triangulation [41].

Interestingly, Pavan et al. [67] performed a recent retrospective multinational multicenter study including 737 adrenalectomies, 36% of whom were performed with LESS technique. Moreover, the authors [67] noted that from 2008 to 2013, LESS had the fastest increase in utilization (6% per year) among the techniques analyzed and that this procedure was mostly performed in Asia and South America.

Both transperitoneal and retroperitoneal approach has been described for Robot-assisted LESS adrenalectomy (see Table 3). Arghami et al. [68] reported a matched-cohort study comparing 16 single-port robot-assisted adrenalectomies with 16 laparoscopic adrenalectomies. They reported the same rate of conversion to open surgery (6%) but in the robot-assisted group, 12% of cases were converted to laparoscopic surgery due to dense inflammatory reaction and to difficulty in visualizing adrenal gland. Further, the same complications rate between the two approaches was described (12%) and there were no peri-operative deaths in both groups. Moreover, the authors [68] found that patients in the robotic-assisted LESS group had statistically significantly lower narcotic use in the first 24 h after surgery.

**Table 3:** Robot-assisted LESS adrenalectomy series

Authors	Cases	Approach (TP/RP)	EBL (ml)	MOT (min)	MTS (cm)	Intra-operative complications	Post-operative complications	Conversions	Re-operations	Mean hospital stay (days)	Mortality
Arghami et al. [68]	19	19/0	576	183	3.6	1 bleeding (5.3%)	1 respiratory distress (5.3%) 1 ileus (5.3%)	3 (15.8%)	0	2.3	0
Lee et al. [69]	38	38/0	393	234	4.9	3 bleeding (7.9%)	4 ileus (10.5%) 2 adrenal failure (5.3%)	7 (18.4%)	0	2.8	0
Park et al. [70]	5	0/5	46	159	1.5	0	0	0	0	4	0
Choi et al. [95]	2	2/0	250	167	2.5	0	0	0	0	3.5	0

*NR* Not Reported, *TP* Trans-peritoneal, *RP* Retro-peritoneal, *EBL* Estimate blood loss, *MOT* Mean operative time, *MTS* Mean tumor size

In a study on 33 patients who underwent robot-assisted single site–site adrenalectomy, Lee et al. [69] reported a conversion rate to laparoscopic surgery of 13% and to open surgery of 6.1%; peri-operative complications rate was 15.8%. In this study [69], it is underlined that that the technical limitations encountered were related to the increased flexibility of the instruments that occurs when they are inserted farther in the trocar. They suggested that this limitation can be outweighed by manipulation of the single-site port to a more cephalad position and with the use of a longer trocar. Furthermore, The authors [69] concluded that Robot-assisted LESS is comparable to the others minimally invasive approach in terms of safety and outcomes and it can be adopted both for non-functional and functional tumors and even in obese patients.

Furthermore, the robotic LESS approach has been performed even with the retroperitoneal access; Park et al. [70] reported their initial experience on five patients with encouraging outcomes (no conversions, no complications). Due to the small operative space, they recommend to start performing robot-assisted LESS with retroperitoneal approach for tumors smaller than 2 cm in patients with a body mass index lower of 30 kg/m^2^ and gradually extending the indications with increasing experience.

Finally, Wang et al. [71] performed a meta-analysis and reported that laparoscopic single-site adrenalectomy appears to be a feasible and safe alternative to conventional LA with a lower post-operative pain, although associated with a longer operative time.

Assumed that safety and effectiveness are key factors in the selection of a technique, the role of robotic system with this approach is still to be defined as large case studies are lacking.

## Indocyanine green fluorescence imaging during robotic adrenalectomy

Endocrine surgery is particularly appropriate for IndoCyanine Green (ICG) fluorescence imaging due to the elevated blood supply of gland parenchyma [72]. ICG fluorescence was first used in humans by Manny et al. [73] in 2013 in a case series of three robot-assisted partial adrenalectomies.

The use of ICG fluorescence enables visual distinction between hyperfluorescent adrenocortical tissue (highly vascular) and hypofluorescent retroperitoneal tissue (poor vascular), making the dissection plan easier to be identified [74]. Furthermore, it resulted helpful in defining the border between tumor and surrounding normal tissue (particularly useful during cortical-sparing adrenalectomy for pheochromocytoma or bilateral procedure) and enables to evaluate the vascularity of the remaining adrenal parenchyma at the end of the dissection [75]. Moreover, the real-time feedback of ICG fluorescence supports to compensate the lack of haptic feedback during RA, traditionally used to determine the margin of resection [74].

Kahramangil et al. [76] in a case series of 100 robot-assisted adrenalectomy reported that the exhibition of fluorescence is depending on the histologic origin (cortical versus medullary); in their study, adrenocortical tumors displayed a higher intensity of fluorescence compared with the surrounding retroperitoneal soft tissues, whereas medullary adrenal tumors were non-fluorescent. This feature could be particularly useful when performing cortical-sparing adrenalectomy for familial bilateral pheochromocytoma [72]. Manny et al. [73] hypothesized that the lack of fluorescence of pheochromocytoma could be related to the lower expression of bilitranslocase, an enzyme affecting ICG uptake.

In a case series of ten RA, Sound et al. [77] reported that ICG imaging resulted helpful with the conduct of the operation in eight out of ten procedures. In two cases, ICG imaging did not add any information; one was a patient who underwent right retroperitoneal adrenalectomy where the fluorescence of the liver did not enable any contrast distinction between the adrenal and retroperitoneal tissues, and one was a patient with a 6.5-cm adrenocortical neoplasm where the adrenal mass did not show any fluorescence.

Further, Colvin et al. [72] in a study conducted on 43 robot-assisted adrenalectomy, reported that the distinction of the adrenal tumor’s border with use of ICG increases the precision of dissection compared to conventional robotic visualization in about half of cases.

In conclusion, although this new technique does not replace an accurate dissection, the use of ICG fluorescence may be useful for patients requiring partial adrenalectomy to avoid adrenal failure and in refractory cases where it is challenging to resect completely the adrenal parenchyma. The loss of tactile feedback proper of the robotic surgery can be partially compensated with this imaging modality; nevertheless, future researches will investigate the impact of ICG fluorescence on outcomes.

## Cost-effectiveness

One of the most significant concerns about robot-assisted surgery regards its prohibitive costs. The elevated cost associated with robotic surgery is primarily related to the purchase and the maintenance of the unit, the high instruments cost, the use of semi-disposable instruments and the longer operative time [15].

Agcaoglu et al. [35] in a comparative study between posterior retroperitoneal RA vs LA reported that the additional cost of robotic surgery (including robotic instruments, drapes and the annual maintenance fee) is about 900–950 $ per procedure. Furthermore, Brunaud et al. [30] in a cost evaluation reported that RA is 2.3 times more costly than LA (€ 4102 vs € 1799). However, cost reduction can be obtained by means of increasing the number of RA per year and with the depreciation of the robotic system [30, 78]. Indeed, in a German study by Probst et al. [79], the additional cost for using the robotic system was € 2288 per procedure, taking into consideration a center with 150 robotic operations per year; this was calculated considering € 400,000 of depreciation cost per year. Since this fee has to be allocated to all robotic procedures, centers with higher number of robotic operations can benefit of a lower cost burden.

Recently, Feng et al. [80] in a comparative study between RA and LA did not observe significantly differences in terms of costs. They suggested that possible ways of lowering costs are limiting unnecessary robotic instruments and energy devices and having an experienced team at the operating table. Anyway, their analysis did not take into account the purchase fee since the decision to buy the robotic system was already made, and this fact weakens a lot their conclusion as the robotic system has a considerable cost (the price ranges from $1 million to $2.5 million for each unit) [81].

Overall, the costs represent the major drawback of RA and significant benefits should be demonstrated to justify the use of robotic system for adrenal lesions, even in high-volume centers.

## Conclusion

In conclusion, LA should be considered the gold standard technique for adrenal lesions. Both transabdominal and retroperitoneoscopic technique have proved to be safe and effective and the approach should be chosen on the basis of surgeon’s experience. Recently, several case series have demonstrated the feasibility and the safety of robot-assisted adrenalectomy in high-volume centers with outcomes comparable to LA. Overall, the robotic system provides some technical advantages, such as increased dexterity, 3-D magnified vision and tremor-filtering technology, but these benefits do not outweigh the elevated cost and the prolonged operative time. Since adrenalectomy is a demolitive procedure that does not require a reconstructive phase, the increased maneuverability provided by the robotic system seems not to be reflected in substantial benefits in terms of outcomes, whereas the concerns regard this technique persist. Notwithstanding, RA may be theoretically well suited for performing cortical-sparing adrenalectomy since this procedure can take advantage of the better visualization and the increased dexterity to effectively preserving the cortical remnant. Moreover, RA could play a role during complex cases in which a reconstructive phase with fine suture is needed.

## Data Availability

Available if requested.
